# Supplementary material for: Cardioprotective effects of AMPK activation in H1N1 influenza virus infection
Source: bioRxiv. 2025 Aug 28:2025.08.28.672931. Preprint. [Version 1] doi: 10.1101/2025.08.28.672931 (PMC12407972; doi:10.1101/2025.08.28.672931)
Supplement: Supplement 1 [file NIHPP2025.08.28.672931v1-supplement-1.pdf]

bioRxiv preprint doi: https://doi.org/10.1101/2025.08.28.672931; this version posted August 28, 2025. The copyright holder for this preprint (which was not certified by peer review) is the author/funder, who has granted bioRxiv a license to display the preprint in perpetuity. It is made available under aCC-BY 4.0 International license.

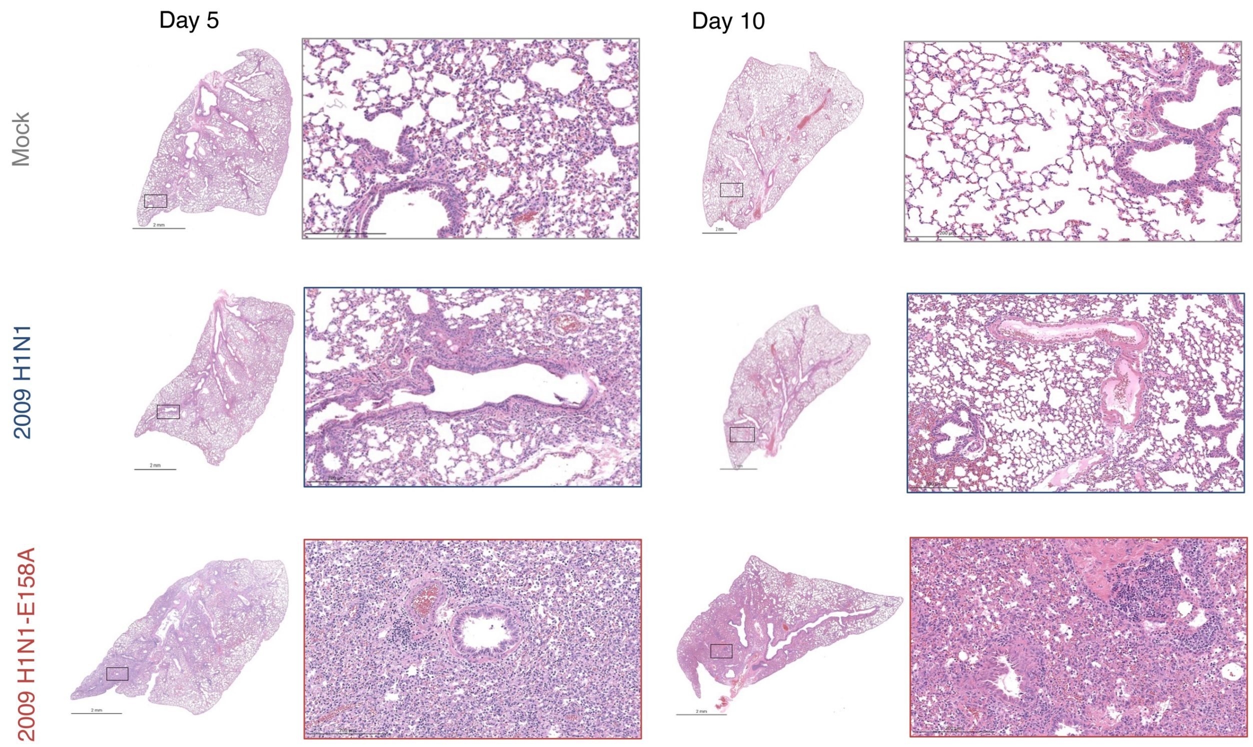

Supp Figure 1: Mouse adapted H1N1-E158A virus robustly induces lung histological damage. Representative H&E-stained lung sections at days 5 and 10 post-infection with 200 TCID50 H1N1 virus or H1N1-E158A virus or mock (PBS) innoculation. Whole-lung sections (left panels; scale bars, 2 mm) and corresponding high-magnification images (right panels; scale bars, 200 µm) are shown for each condition and time point. Boxes in whole-lung images indicate the regions displayed at higher magnification. Note: Day 10 unzoomed images are repeated from main text Figure 1D.

bioRxiv preprint doi: https://doi.org/10.1101/2025.08.28.672931; this version posted August 28, 2025. The copyright holder for this preprint (which was not certified by peer review) is the author/funder, who has granted bioRxiv a license to display the preprint in perpetuity. It is made available under aCC-BY 4.0 International license.

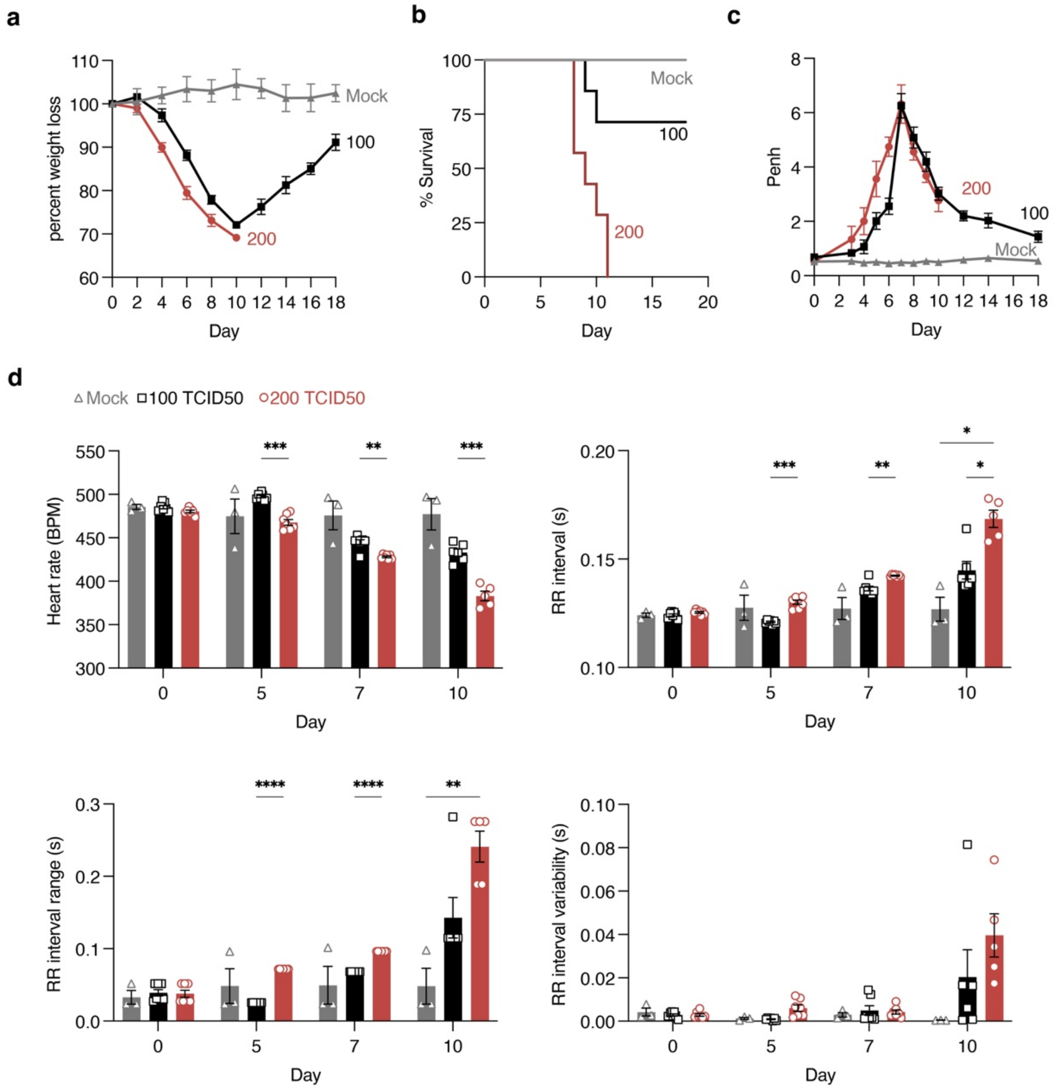

Supp Figure 2: Viral dose responsiveness of lung and heart functional readouts. Mice were intranasally infected with 100 or 200 TCID50 of H1N1-E158A virus or were mock (PBS) innoculated. **a,** Body weight loss following infection. Each data point represents the average weight of individual mice normalized to 100% of their starting weight on day 0. Error bars indicate SEM. **b,** Kaplan-Meier survival curves. **c,** Whole-body plethysmography analysis of Penh as an indicator of respiratory dysfunction. Each data point represents the average Penh value from multiple mice; error bars indicate SEM. Mice in **a,b,c** were monitored daily for 18 days. Group sizes: Mock, n = 3; H1N1, n = 7; H1N1-E158A, n = 7. **d,** Heart rate, RR interval, RR interval range, and RR interval variability in Mock and H1N1-E158A infected mice at baseline and at days 5, 7, and 10 post-infection. Group sizes: Mock, n = 3 (all time points); H1N1-E158A (100 TCID50): n = 7 (baseline, days 5 and 7), n = 6 (day 10); H1N1-E158A (200 TCID50): n = 7 (baseline, days 5 and 7), n = 5 (day 10). All data are from a single experiment. Statistical analysis by two-way ANOVA with Sidak's multiple comparisons test; *P < 0.05, **P < 0.01, ***P < 0.001, ****P < 0.0001.

bioRxiv preprint doi: https://doi.org/10.1101/2025.08.28.672931; this version posted August 28, 2025. The copyright holder for this preprint (which was not certified by peer review) is the author/funder, who has granted bioRxiv a license to display the preprint in perpetuity. It is made available under aCC-BY 4.0 International license.

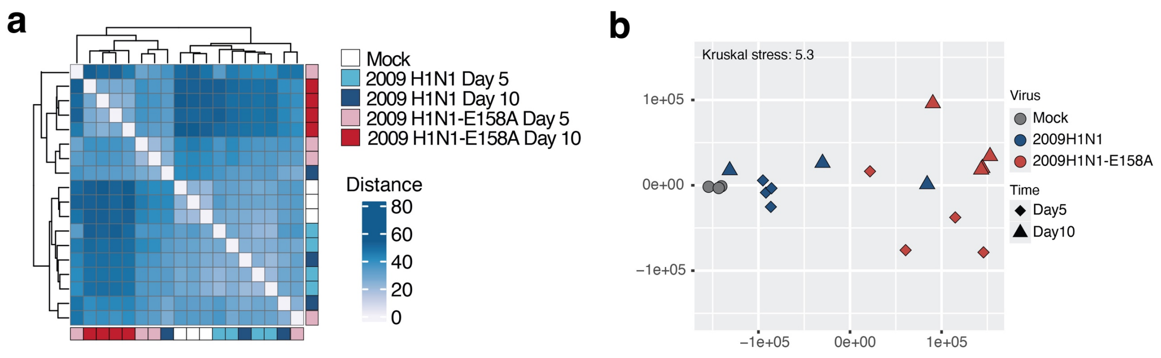

Supp Figure 3: RNA sequencing gene expression correlation and principal components analysis for individual samples. Bulk RNA sequencing was performed on heart samples from mice infected with 200 TCID50 H1N1 virus or H1N1-E158A virus in comparison to mock (PBS) inoculated animals. **a,** Heatmap showing the correlation of gene expression profiles across all samples. The color scale indicates the degree of similarity between samples. **b,** Multidimensional scaling (MDS) representation of the heart transcriptional responses elicited by H1N1 and H1N1-E158A influenza virus strains at days 5 and 10. Each data point represents a biological replicate, where color denotes viral infection and shape indicates timepoint post infection. The quality of the representation is provided by the Kruskal stress value, with a low percentage of Kruskal stress (5.3%).

bioRxiv preprint doi: https://doi.org/10.1101/2025.08.28.672931; this version posted August 28, 2025. The copyright holder for this preprint (which was not certified by peer review) is the author/funder, who has granted bioRxiv a license to display the preprint in perpetuity. It is made available under aCC-BY 4.0 International license.

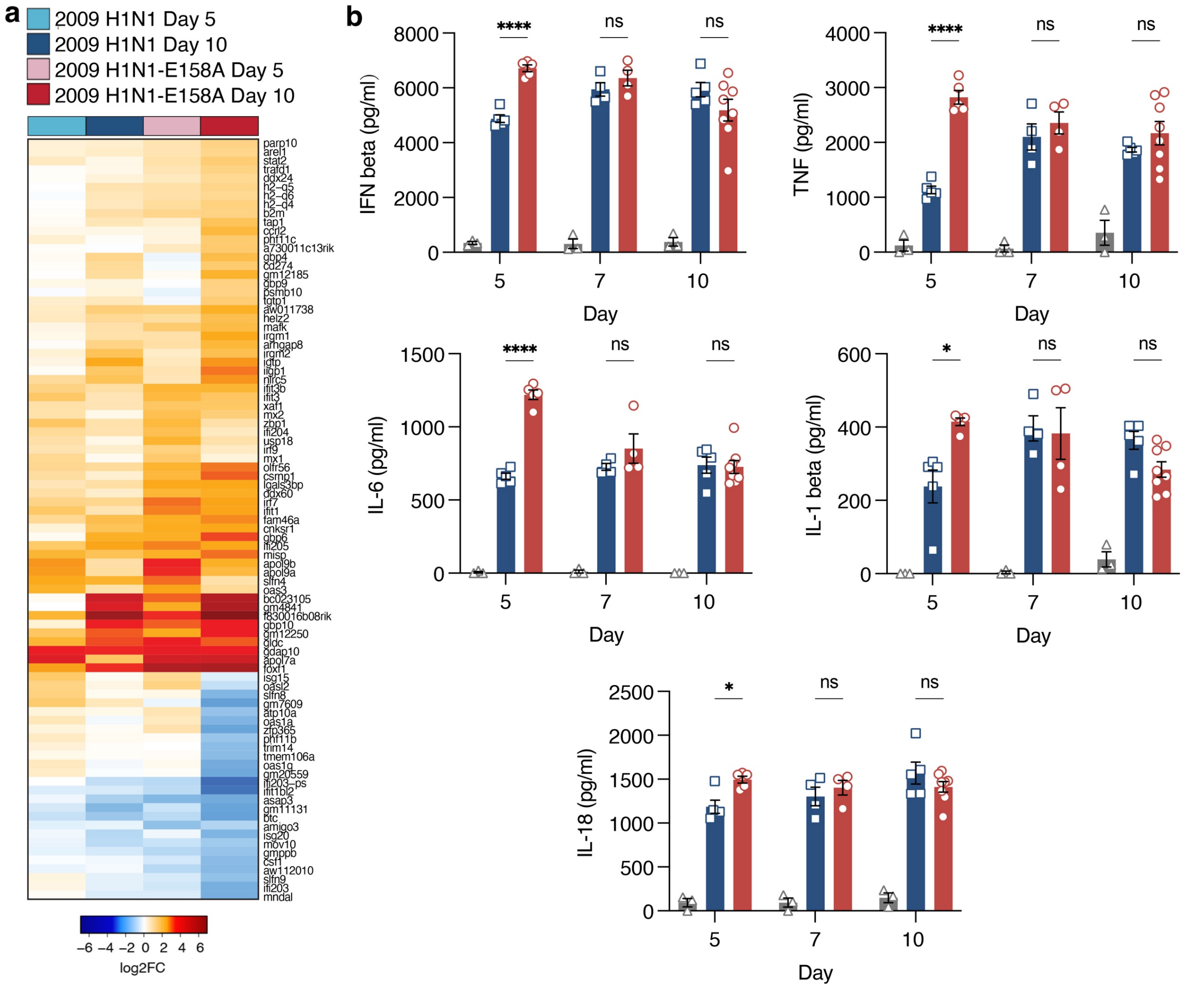

Supp Fig 4: Mouse adapted 2009 H1N1 virus induces cardiac antiviral and inflammatory responses. a, Bulk RNA sequencing data from infected mice relative to mock controls (as in Fig. 4) were analyzed to generate a heat map of Heatmap of 87 interferon-stimulated genes (ISGs) identified from the union of differentially expressed genes across experimental groups. **b,** Quantification of IFN-β, TNF, IL-6, IL-1β, and IL-18 levels in heart homogenates from mice inoculated with PBS (Mock) or 200 TCID50 H1N1 virus, or H1N1-E158A virus, measured by ELISA at days 5, 7, and 10 post-infection. Each data point represents a single mouse; Error bars indicate SEM. Group sizes: Mock (days 5, 7, and 10), n = 3; H1N1: day 5, n = 5; day 7, n = 4; day 10, n = 5; H1N1-E158A: day 5, n = 5; day 7, n = 4; day 10, n = 8. Data are from a single experiment. Statistical significance was determined by two-way ANOVA with Tukey's multiple comparisons test; *P < 0.05, ****P < 0.0001.

bioRxiv preprint doi: https://doi.org/10.1101/2025.08.28.672931; this version posted August 28, 2025. The copyright holder for this preprint (which was not certified by peer review) is the author/funder, who has granted bioRxiv a license to display the preprint in perpetuity. It is made available under aCC-BY 4.0 International license.

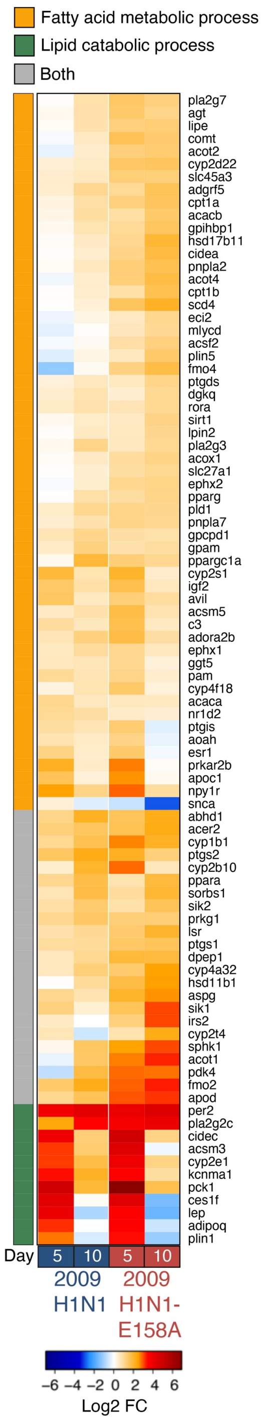

Supp Fig 5: RNA sequencing reveals alteration of lipid metabolism-associated genes in influenza virus infected hearts. Heat map showing the relative expression of genes involved in cardiac lipid metabolism and mitochondrial pathways at days 5 and 10 post-infection across experimental groups.

bioRxiv preprint doi: https://doi.org/10.1101/2025.08.28.672931; this version posted August 28, 2025. The copyright holder for this preprint (which was not certified by peer review) is the author/funder, who has granted bioRxiv a license to display the preprint in perpetuity. It is made available under aCC-BY 4.0 International license.

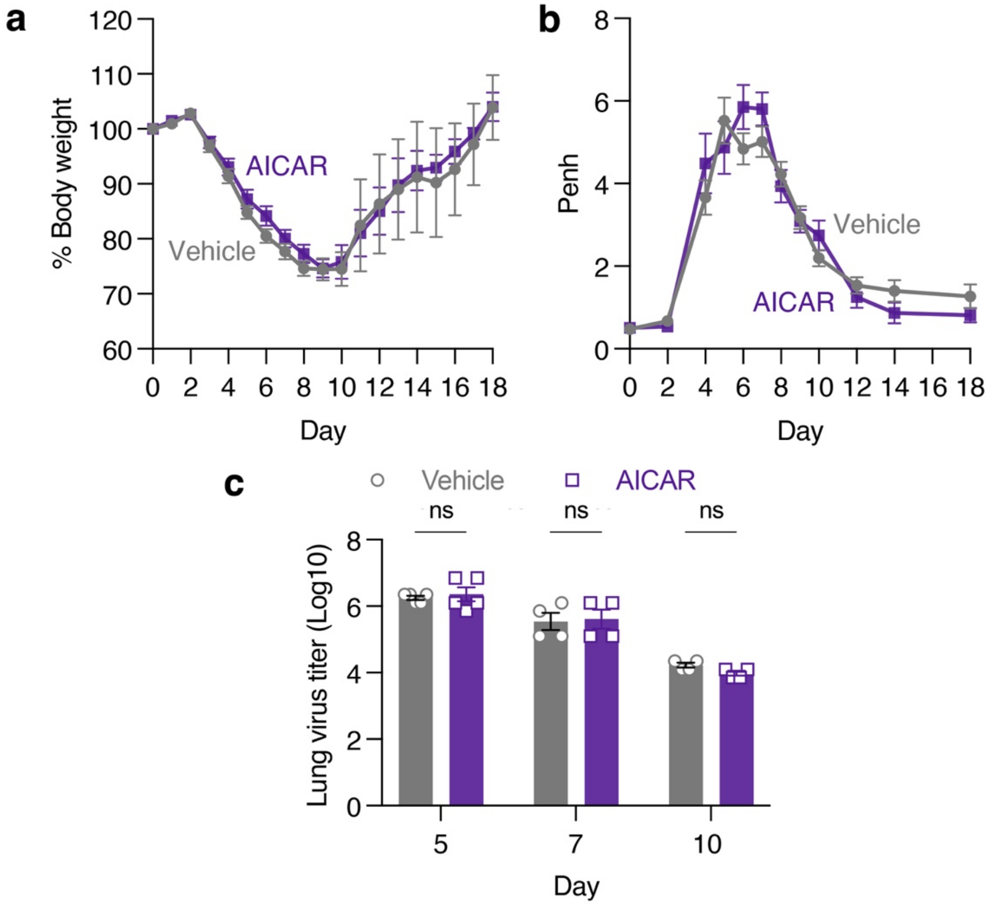

Supp Fig 6: AICAR treatment does not affect weight loss, lung function, or lung viral titer in influenza virus infection. Mice infected with H1N1-E158A (200 TCID50) were treated daily with AICAR (100 mg/kg in saline) or vehicle (saline) from day 4 through day 10 post-infection. **a,** Body weight loss over time following infection. Each data point represents the average weight of individual mice normalized to 100% of baseline (day 0); error bars indicate SEM. Data from two experiments (Vehicle: n = 20; AICAR-treated: n = 16). **b,** Whole-body plethysmography measurements of PenH as a marker of respiratory function. Each data point represents the average Penh value from multiple mice; error bars indicate SEM. Data from two experiments (Vehicle: n = 20; AICAR-treated: n = 16). **c,** Lung viral titers at days 5, 7, and 10 post-infection in H1N1-E158A infected mice treated with AICAR or saline (vehicle). Data are from a single experiment. Group sizes: AICAR and vehicle groups, day 5, n = 5; days 7 and 10, n = 4. Statistical analysis was evaluated by two-way ANOVA followed by Sidak's multiple comparisons test.

bioRxiv preprint doi: https://doi.org/10.1101/2025.08.28.672931; this version posted August 28, 2025. The copyright holder for this preprint (which was not certified by peer review) is the author/funder, who has granted bioRxiv a license to display the preprint in perpetuity. It is made available under aCC-BY 4.0 International license.

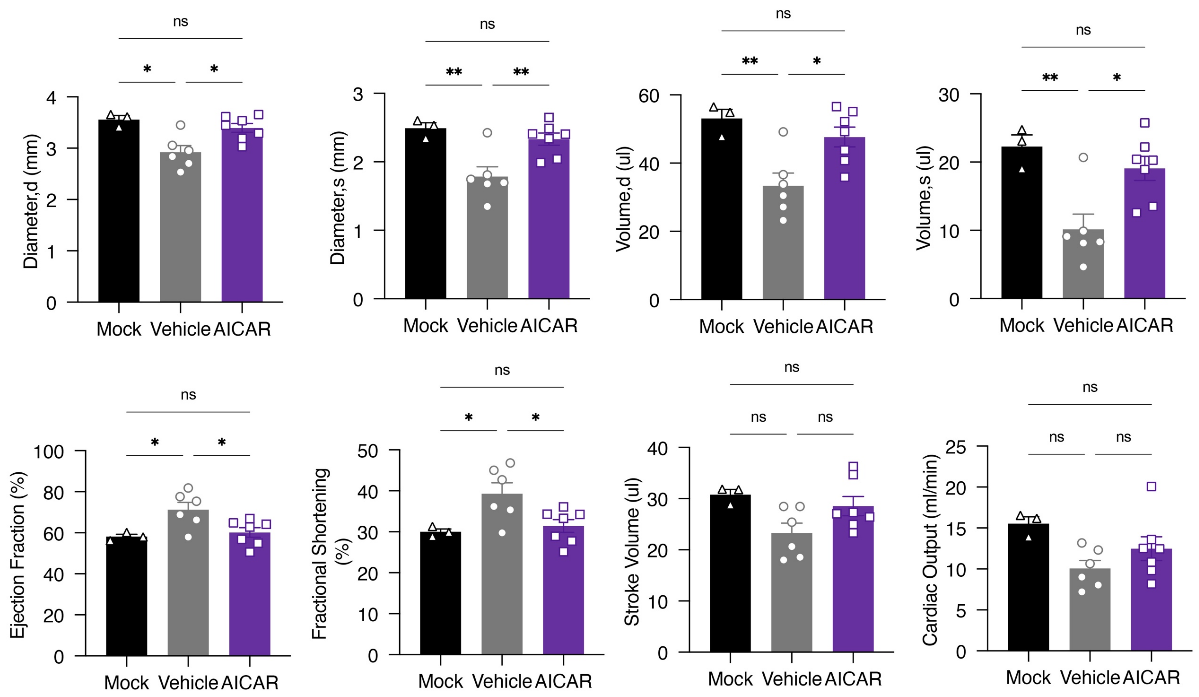

Supp Fig 7: AICAR treatment normalizes key echocardiography parameters in influenza virus infected mice. Mice infected with H1N1-E158A (200 TCID50) were treated daily with AICAR (100 mg/kg in saline) or vehicle (saline) from day 4 through day 10 post-infection. Cardiac morphology and function were evaluated by 2-D echocardiography at 10 days post infection or on mock infected animals. Standard echocardiography readout data are plotted with each dot corresponding to an individual mouse. Error bars indicate SEM (Pre-infection: Mock n = 3, Vehicle n = 6, AICAR n = 7; Statistical significance was determined by one-way ANOVA with Tukey's multiple comparisons test; *P < 0.05, **P < 0.01.
